# Neurologically predominant fat embolism syndrome in an octogenarian with dementia: diagnostic challenges and prolonged recovery

**DOI:** 10.1186/s12883-026-04740-9

**Published:** 2026-02-19

**Authors:** Shota Fukaura, Kentaro Hori, Shingo Kawakami, Takashi Katayama, Yoko Suzuki

**Affiliations:** 1 Department of Neurology, Omori Red Cross Hospital, l-30-4 Chuo, Ota-ku, Tokyo, 143-8527 Japan; 2 Department of Radiology, Omori Red Cross Hospital, Tokyo, Japan

**Keywords:** Fat embolism syndrome, Neurological-predominant, Older adults, Magnetic resonance imaging, Diffusion-weighted imaging, Susceptibility-weighted imaging, Case report

## Abstract

**Background:**

Neurologically predominant fat embolism syndrome (FES) without respiratory involvement is very rare in elderly patients with pre-existing dementia. Diagnosis is challenging as symptoms may be misattributed to delirium.

**Case presentation:**

We report the case of an 82-year-old man with dementia with Lewy bodies who developed progressive altered consciousness following a femoral neck fracture. Despite the absence of respiratory symptoms, brain magnetic resonance imaging revealed the characteristic findings of cerebral fat embolism, including multiple hyperintense lesions on diffusion-weighted imaging(DWI) and numerous hypointense lesions on susceptibility-weighted imaging(SWI) of the corpus callosum, cerebellum, and cerebrum. The patient’s clinical stability indicated conservative management was appropriate. Comprehensive supportive care included enteral nutrition, prevention of immobility-related complications, and rehabilitation. Corticosteroid therapy was not indicated. Gradual neurological improvement occurred over 84 days, with the patient achieving partial functional recovery including functional communication and mobility. Follow-up MRI at 2 months showed persistent microhemorrhages with minimal interval change, despite significant clinical improvement.

**Conclusions:**

This case highlights the diagnostic challenges of neurologically predominant FES in elderly patients with dementia, where symptoms may easily be confused with hypoactive delirium. Advanced MRI sequences (DWI and SWI) are essential for diagnosis. Despite advanced age and pre-existing cognitive impairment, prolonged neurological recovery is possible with sustained supportive care, emphasizing the importance of avoiding premature therapeutic nihilism in this vulnerable population.

## Background

Fat embolism syndrome (FES) is an infrequent but potentially life-threatening complication that follows the intravasation of marrow fat into the systemic circulation, most often after long bone or pelvic fractures. This condition is seen most often after fractures of the femoral shaft or neck [1]. Neurological manifestations typically occur 12–72 h after injury and range from mild confusion to coma or focal deficits [2, 3]. FES classically presents with the triad of respiratory insufficiency, neurological impairment, and petechial rash [4]. While pulmonary dysfunction occurs in the majority of patients (75%) [2], neurologically predominant FES without respiratory involvement represents a rare clinical presentation [5, 6]. In elderly patients with pre-existing cognitive impairment, diagnosis is particularly challenging. Systematic review of 268 cases showed a mean age of 33 years [7], highlighting the rarity of this condition in octogenarians. Cerebral fat embolisms (CFE) can result in microinfarcts, vasogenic oedema, and petechial haemorrhage, which are best visualised using advanced magnetic resonance imaging (MRI) techniques [4]. Herein, we present the case of an 82-year-old man with dementia with Lewy bodies who developed neurologically predominant FES following a femoral neck fracture, demonstrating the diagnostic utility of advanced MRI sequences and the potential for prolonged neurological recovery despite advanced age and pre-existing cognitive impairment.

## Case presentation

An 82-year-old man sustained a femoral neck fracture after falling in March 2025. Prior to the fracture, the patient was residing in a care facility. He was able to ambulate independently. He had a pre-existing diagnosis of dementia with Lewy bodies. The following morning, he developed progressively impaired consciousness during breakfast and was admitted to a local hospital. Despite supportive care, the cause of his altered mental status remained unclear. Four days later, he was transferred to our hospital for further evaluation.

**Fig. 1:**
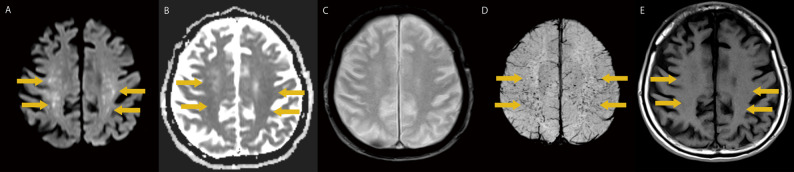
Brain MRI obtained 7 days after symptom onset demonstrating characteristic findings of cerebral fat embolism and superior sensitivity of SWI. **A** DWI shows multiple small hyperintense lesions (arrows) distributed bilaterally in the cerebral white matter representing cytotoxic edema caused by fat emboli. The lesions demonstrate the characteristic "starfield pattern" with multifocal distribution crossing multiple arterial territories. **B** ADC map at a representative level shows restricted diffusion (hypointense signal, arrows) in the affected areas, confirming acute cytotoxic edema. **C** Conventional T2*-weighted imaging at a representative level shows no detectable microhemorrhages, appearing essentially normal. **D** SWI at a representative level reveals numerous hypointense lesions (arrows) representing microhemorrhages, with the characteristic distribution pattern involving the cerebral white matter. Note the marked discrepancy between T2* (panel **C**, negative) and SWI (panel **D**, extensive abnormalities), demonstrating the superior sensitivity of SWI for detecting cerebral microhemorrhages in fat embolism. **E** Pre-contrast T1-weighted imaging at a representative level shows subtle hypointensity (arrows) in regions corresponding to the DWI hyperintensities (panel **A**). No T1 hyperintensity is observed, indicating that individual fat globules are too small to be directly visualized despite their presence as evidenced by microhemorrhages on SWI (panel **D**)

Upon admission, the patient had a Glasgow Coma Scale score of E3V1M4. Focal neurological deficits were not observed. Laboratory investigations revealed: white blood cell count 7,200/µL, platelet count 190,000/µL, hemoglobin 10.8 g/dL, and D-dimer 8.6 µg/mL. Pelvic computed tomography (CT) confirmed a femoral neck fracture. Head MRI revealed multiple small hyperintense lesions on diffusion-weighted imaging (DWI) with corresponding restricted diffusion on apparent diffusion coefficient (ADC) maps, representing cytotoxic edema. Notably, conventional T2*-weighted imaging failed to detect microhemorrhages, whereas susceptibility-weighted imaging (SWI) demonstrated numerous small hypointense lesions diffusely involving the corpus callosum, cerebellum, and cerebral white matter—findings consistent with cerebral fat embolism (Fig. 1). Cerebrospinal fluid analysis revealed a normal cell count, normal protein concentration, normal interleukin-6 level, and IgG index, which helped to exclude infectious or inflammatory aetiologies. Electroencephalogram demonstrated generalised slow-wave activity (approximately 6 Hz) without epileptiform discharges, consistent with diffuse cerebral dysfunction. Therefore, FES was favoured as the diagnosis of exclusion. Despite the absence of any respiratory manifestations and normal admission chest CT without pulmonary opacities, the patient developed cerebral signs consistent with the neurologically predominant FES subtype. Transthoracic echocardiography revealed no findings of patent foramen ovale. Transesophageal echocardiography (TEE) was not performed; therefore, right-to-left shunt was not definitively excluded.

Considering the lack of evidence-based therapies for FES, supportive management strategies have been adopted. Enteral nutrition was provided via a nasogastric tube and the patient was closely monitored for neurological recovery. No corticosteroids were administered and oxygen supplementation was not required.

On hospital day 17, the first signs of recovery were noted when the patient began to vocalise (“ah ah ah”) in response to verbal stimuli and opened his eyes with visual tracking. By day 33, he was able to follow simple verbal commands (e.g., “raise your hand”) and state his own name. Spontaneous smiling was observed on day 37. Thereafter, progressive improvements in consciousness and functional abilities persisted, allowing for the initiation of oral feeding and rehabilitation therapies.

**Fig. 2:**
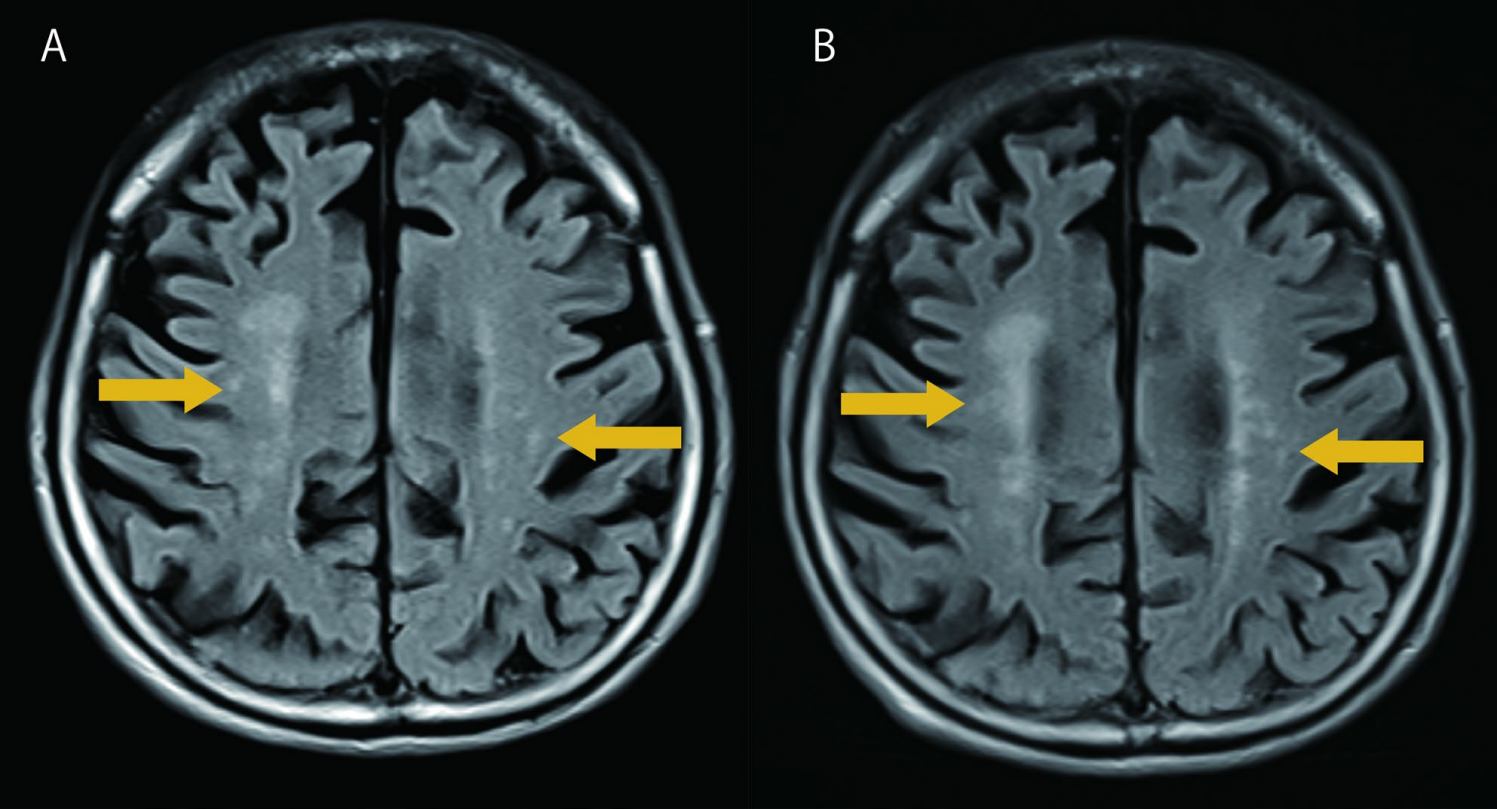
Serial FLAIR imaging demonstrating imaging-clinical dissociation in cerebral fat embolism. **A** Baseline FLAIR images obtained 7 days after symptom onset show multiple hyperintense lesions in the bilateral cerebral white matter, corpus callosum, and cerebellum. **B** Follow-up FLAIR images at 2 months show minimal interval change with persistent structural abnormalities in the same regions

Follow-up brain MRI performed approximately 2 months after symptom onset showed minimal interval change on fluid-attenuated inversion recovery (FLAIR) imaging, with persistent structural abnormalities in the white matter, corpus callosum, and cerebellum (Fig. 2). On day 84 of hospitalisation, the patient was transferred to a rehabilitation facility. At the time of transfer, the patient was able to maintain a seated position in a wheelchair and engage in simple verbal communication. There was no evidence of aphasia or limb paralysis. Swallowing function had recovered remarkably well, with the patient demonstrating smooth swallowing mechanics and good food recognition, although the quantity of oral intake remained limited. Comprehensive assessment of higher cortical function is limited by a preexisting dementia with Lewy bodies. The femoral neck fracture was not surgically treated, which contributed to the patient’s limited mobility at discharge. The patient achieved partial functional recovery including verbal communication and wheelchair mobility.

## Discussion

This case report describes a rare presentation of neurologically predominant FES in an older adult patient and highlights several important clinical and diagnostic considerations.

FES typically presents with a classic triad of respiratory, neurological, and dermatological manifestations. However, neurologically predominant variants without respiratory involvement have been increasingly recognised, particularly in younger patients, following long bone fractures [8, 9]. In elderly patients with pre-existing cognitive impairment, this presentation poses significant diagnostic challenges. In this clinical scenario, the most common diagnosis would be hypoactive delirium, which frequently occurs in elderly patients following orthopedic trauma. Other common considerations include progression of underlying dementia, metabolic disturbances, infection, or stroke. Cerebral fat embolism is rarely considered in such cases, particularly when respiratory symptoms are absent. In elderly patients with dementia, altered consciousness may be hastily attributed to hypoactive delirium or disease progression without adequate investigation [10]. Several features in our patient prompted further investigation: the temporal relationship to fracture (symptoms began within 24 h), absence of metabolic or infectious causes, and lack of response to supportive care. These atypical features led to advanced MRI, which revealed the characteristic findings of cerebral fat embolism. This case underscores the importance of maintaining a high clinical suspicion of FES in elderly patients presenting with altered mental status following bone fractures, even in the absence of respiratory symptoms.

Advanced MRI sequences, particularly SWI, proved essential for establishing the diagnosis in our case. Our case demonstrates an important diagnostic discrepancy between T2* and SWI sequences that has critical clinical implications. Conventional T2*-weighted imaging failed to reveal any microhemorrhages, whereas SWI clearly demonstrated numerous hypointense lesions with the characteristic distribution pattern involving the corpus callosum, cerebellum, and cerebral white matter. This observation corroborates earlier evidence demonstrating superior sensitivity of SWI over conventional T2* imaging, with prior reports indicating that only a subset of lesions visible on SWI can be detected on conventional T2*-weighted images [11]. Despite this evidence, more recent literature has described these sequences as having equivalent diagnostic capability for detecting microbleeds in CFE [12]. Our case provides important confirmatory evidence supporting the earlier findings [11], as T2* imaging was completely negative despite extensive microhemorrhages visible on SWI. Combined with the characteristic “starfield pattern” on DWI, the SWI findings provided definitive diagnostic evidence. This finding has important clinical implications. In patients with suspected FES following orthopedic trauma, reliance on conventional T2*-weighted imaging alone may result in false-negative results and diagnostic delay. Our experience reaffirms that SWI should be considered an essential—rather than optional—component of neuroimaging protocols when FES is suspected, particularly in clinically equivocal cases where prompt diagnosis is crucial. The characteristic distribution pattern observed in our patient provides insights into the pathophysiology of cerebral fat embolism. Unlike typical arterial embolic strokes that follow defined vascular territories, the lesions in our patient demonstrated bilateral, multifocal distribution independent of arterial territories. DWI hyperintensities were observed bilaterally in the subcortical white matter, corpus callosum, and cerebellum—regions that span multiple arterial territories. This characteristic bilateral, multifocal, territory-independent distribution pattern has been well documented in cerebral fat embolism and is thought to reflect the mechanism of numerous small fat globules simultaneously entering the cerebral circulation and lodging in small vessels throughout the brain, rather than occluding a single major arterial branch [12].

Our patient showed isolated neurological symptoms without respiratory problems or hemodynamic instability. This represents the neurologically predominant variant of FES. Recovery was gradual, taking 84 days, with first signs appearing on day 17. Armstrong et al. studied 34 patients with cerebral fat embolism, mostly young adults (mean age 29.7 years, range 18–70 years). In their study, 96.3% of survivors recovered completely within an average of 4.7 weeks (range 2–13 weeks). Three patients recovered after 8 weeks; these late-recovery cases were all young adults (ages 19–24 years) [13]. Recovery in our 82-year-old patient took 84 days, much longer than the younger patients in Armstrong’s study (average 4.7 weeks). Despite his age and pre-existing dementia, he achieved functional improvement with sustained supportive care. While we cannot generalize from a single case, this experience suggests that functional recovery is possible in elderly patients with cerebral fat embolism, even when the recovery course is prolonged. This observation supports continued supportive care in elderly patients rather than premature assumptions about recovery potential based on age alone.

Follow-up MRI at 2 months demonstrated important temporal changes with significant imaging-clinical dissociation. DWI showed pseudonormalization— reflecting the natural evolution of cytotoxic edema beyond the visible phase rather than indicating tissue recovery. In contrast, T2-weighted and FLAIR images showed minimal interval change, and SWI continued to demonstrate persistent microhemorrhages due to hemosiderin deposition. Despite these persistent structural abnormalities on T2/FLAIR and SWI, the patient demonstrated meaningful functional improvement including wheelchair mobility and verbal communication. This underscores that clinical assessment of recovery should not rely solely on imaging resolution, as neurological improvement can occur despite permanent structural changes visible on MRI.

Management of neurologically predominant FES without systemic involvement is primarily supportive. Our patient’s clinical stability—absence of respiratory involvement, hemodynamic stability, normal oxygen saturation— indicated that supportive care was appropriate. The role of corticosteroids in FES remains controversial and lacks high-quality evidence. While some case reports describe potential benefit in severe respiratory FES with refractory hypoxemia, there is no evidence supporting corticosteroid use in neurologically predominant FES without respiratory compromise. Pharmacological intervention was therefore not indicated based on our patient’s clinical presentation.

A limitation of our study was the lack of shunt testing. TTE showed no patent foramen ovale, but we did not perform the TEE; therefore, a right-to-left shunt in the heart or lungs could not be excluded. Importantly, previous reports have described CFE even when TEE is negative for a shunt, indicating that a detectable paradoxical shunt is not required in every case [14]. This uncertainty does not change our diagnosis of cerebral FES but limits conclusions about the exact route of embolisation.

In conclusion, this case emphasizes three key clinical messages. First, consider cerebral fat embolism in elderly patients who develop altered consciousness after long bone fractures, even when respiratory symptoms are absent and even in patients with pre-existing dementia. Do not hastily attribute altered mental status to hypoactive delirium when clinical features are atypical. Second, use advanced MRI sequences—specifically DWI and SWI—when microhemorrhage detection is clinically important. SWI is generally recognized as more sensitive than conventional T2* gradient-echo sequences for detecting microhemorrhages across various clinical conditions, not limited to cerebral fat embolism. Our case demonstrates the critical importance of this difference in cerebral fat embolism: conventional T2* was completely negative, whereas SWI revealed extensive microhemorrhages that were essential for diagnosis. Based on this observation, SWI should be included in the imaging protocol when microhemorrhage detection is relevant. Third, meaningful functional recovery occurred in our 82-year-old patient with pre-existing dementia despite prolonged recovery time. This suggests that sustained supportive care remains important in elderly patients, and recovery potential should not be dismissed based solely on advanced age.

## Data Availability

The datasets used in this study are available from the corresponding author upon request.

## Abbrevations

FES: Fat embolism syndrome
CFE: Cerebral fat embolisms
MRI: Magnetic resonance imaging
CT: Computed tomography
DWI: Diffusion-weighted imaging
SWI: Susceptibility-weighted imaging
TEE: Transoesophageal echocardiography
